# Supplementary material for: Deletion of wfs1 Impairs Oligodendrocyte Precursor Cells Dorsal Distribution and Myelination Through the wfs1-hmgcs1 Axis in Zebrafish
Source: Biology (Basel). 2026 Aug 16;15(16):1402. doi: 10.3390/biology15161402 (PMC13510151; doi:10.3390/biology15161402)
Supplement: Supplementary file 1 [file biology-15-01402-s001.zip › File S1-Transcriptome sequencing data and differentially expressed genes (DEGs) in wfs1-- zebrafish at 5 dpf/2.KEGG/wfs1KOvsWT/ALL/src/dre03050.html]

dre03050


- dre:796450

- Up regulated genes

796450\_1(4.65392097163098)

- Down regulated genes

796450(-1.32752912951059)

- dre:326884

- Up regulated genes

326884\_1(2.09414851723725)

- dre:436816

- Up regulated genes

436816(1.29106339725002)

- dre:30387

- Up regulated genes

30387(2.93847517413541)

Close
